# The effectiveness of RENAL nephrometry score in ablated renal tumors via radiofrequency ablation or cryoablation

**DOI:** 10.3906/sag-1811-131

**Published:** 2019-06-18

**Authors:** Seda ALADAĞ KURT, Cem YÜCEL, Suna ÖZHAN OKTAR, Gonca ERBAŞ, Sinan SÖZEN, Ali Yusuf ÖNER

**Affiliations:** 1 Department of Radiology, Van Training and Research Hospital, Van Turkey; 2 Department of Radiology, Faculty of Medicine, Gazi University, Ankara Turkey; 3 Department of Urology, Faculty of Medicine, Gazi University, Ankara Turkey

**Keywords:** Radiofrequency ablation, cryotherapy, RENAL nephrometry score

## Abstract

**Background/aim:**

This study aimed to evaluate the clinical efficacy of radiofrequency ablation (RFA) and cryotherapy and to scrutinize the therapeutic success of the RENAL (radius, exophytic/endophytic, nearness to collecting system, anterior/posterior, and location) nephrometry score in terms of possible complications and the predictive status of oncological results.

**Materials and methods:**

Forty-five patients with biopsy-proven renal cell carcinomas (32 males, 13 females) treated with RFA and cryotherapy were included. Patients were 22–90 years old (average: 59.2 years). Statistical analyses were performed using SPSS for Windows.

**Results:**

A total of 79 lesions with dimensions varying between 0.9 and 4.5 cm (average: 2.2 cm) were ablated. Complete ablation was achieved for 72 (91.1%) lesions. Six repeat RFA sessions were applied for 4 (5%) lesions with residue/recurrence. The average RENAL nephrometry scores of lesions that underwent complete ablation and those that developed residue/recurrence were 6.3 and 7.7, respectively. The average recurrence-free survival time was 34.8 months (range: 3–55 months), while it was 44.6 months (range: 6–55 months) for cryotherapy and 28.6 months (range: 3–50 months) for RFA.

**Conclusions:**

Ablative therapies are minimally invasive and effective methods for treating small renal tumors. RENAL nephrometry scoring is a valuable system for standardizing renal tumors and evaluating the success of ablative therapies, possible complications, and oncological results.

## 1. Introduction

As the common use of ultrasonography (US) and cross-sectional imaging methods such as computed tomography (CT) and magnetic resonance imaging (MRI) has intensified, it has become increasingly common to identify small renal masses incidentally [1]. Recent developments in medicine over the last few decades have increased the use of surgical and radiological interventions. Due to the high rate of survival in patients with localized renal cell tumors, minimally invasive ablative treatment methods are of interest as potential therapeutic methods. The most popular methods are radiofrequency ablation (RFA) and cryotherapy [2,3].

Ablative therapies, in addition to treating renal masses, including liver, lung, bone, and soft tissue lesions, have a wide range of clinical use with increasing number of indications [4].

In RFA, the target lesion is ablated with coagulation necrosis using heat generated from high-frequency alternating current obtained by uninsulated electrode tips [5,6].

In cryotherapy, during the sudden freezing process, secondary oxidative phosphorylation of ice crystals, cytotoxic oxygen radicals, and finally development of cellular hypoxia destroy the cellular membrane. Necrosis and irreversible destruction that occur due to repetitive freeze-thaw cycles provide the eradication of the tumor [7].

Local ablative therapeutic methods are repeatable and easily applicable. Associated morbidity and mortality rates are lower and the cost of treatment is reduced compared to other surgical therapies. Moreover, these methods can be synchronously monitored, and if necessary used in combination with other therapeutic methods to increase treatment efficacy [3,8]. The main advantage of these methods is better preservation of renal parenchymal volume [9].

The application of quantitative metrics to renal mass ablation has generally been successful to date [10–12]. Therefore, in the present study we used the RENAL (radius, exophytic/endophytic, nearness to collecting system, anterior/posterior, and location) nephrometry scoring system for this purpose. We evaluated the short- to mid-term clinical efficacy of RFA and cryotherapy for the treatment of small renal tumors among selected cases and scrutinized the therapeutic success of the RENAL nephrometry score in terms of possible complications and predictive status of oncological results.

## 2. Materials and methods

A total of 45 patients (32 males and 13 females) were referred to our clinic for ablative therapy. Mean ± SD patient age was 59.2 ± 15.7 (22–90) years. After the Institutional Review Board’s approval, informed consent was obtained from all patients. The study was approved by the Ethics Committee of the Gazi University Faculty of Medicine (Date: 15/06/2011, Decision number: 244).

The main indication for ablative therapies in our series was comorbidity, including advanced age, hypertension, diabetes mellitus, and some other malignancies. Other indications were solitary kidney, patient preference, hereditary tumor, chronic renal failure, and previous renal surgery.

Patients with a platelet count below 50,000/mm3 and INR value above 1.25 were excluded from the study.

At the planning stage, the appropriate ablative treatment method was determined with the use of US and CT/MRI, and tumors were evaluated based on number and dimension of lesions, as well as their localization, accessibility, and proper administration of injection entry. The RENAL nephrometry scoring process developed by Kutikov and Uzzo [10] was used for objective assessment. The score consists of evaluations of the following tumor features: radius (at the maximal diameter), exophytic/endophytic properties, nearness of its deepest portion to the collecting system or sinus, anterior/posterior descriptor, and location relative to polar line. The suffix ‘x’ is used for an unknown anterior/posterior designation, while ‘h’ is used to designate a hilar tumor location (abutting the main renal artery or vein). All components except for the anterior descriptor are scored on a 1-, 2-, or 3-point scale.

All patients underwent biopsy just before the ablation procedure. In 4 patients who were followed up with von Hippel–Lindau disease with multiple lesions, biopsy was applied to a single lesion, with the largest lesion being selected.

Percutaneous procedures were performed under the guidance of US and/or CT, while intraoperative procedures were performed with the guidance of US only.

Similar to the standards specified by Clark et al., in our study the primary approach in exophytic lesions was percutaneous therapy. An intraoperative approach was preferred in patients with multiple lesions, and especially for anterior and centrally located lesions [13]. 

We chose primarily cryotherapy and a laparoscopic approach in some patients to avoid more parenchymal damage in the lesions located in the anterior of the kidney. Cryotherapy was the first treatment method to be preferred by using the cryoresistant feature of the collecting system in centralized lesions [3,14]. 

RFA was the first choice for solid and exophytic lesions. In addition to the convenience of approach in posterior or lateral lesions, another advantage of RFA is its easy applicability with one probe instead of a large number of probes as in cryotherapy.

**Figure 1:**
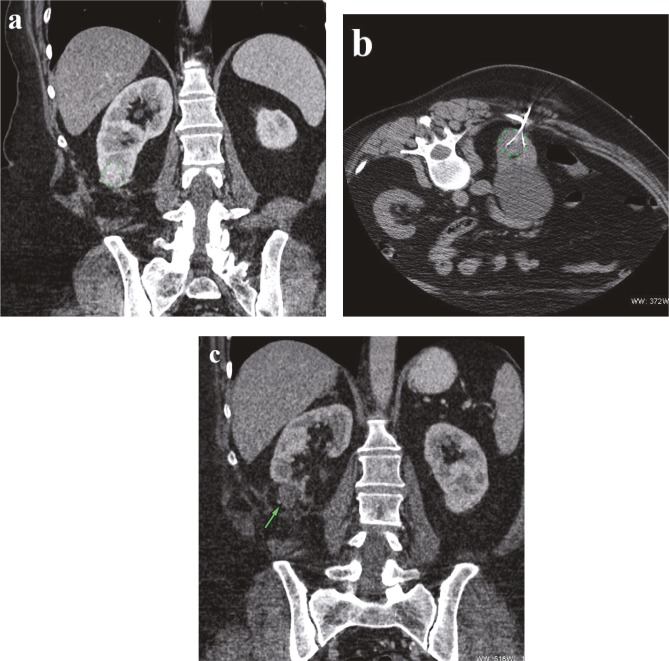
a) Fifty-one-year-old woman with 2.5-cm biopsy-proven renal cell carcinoma marked with green dots. RENAL nephrometry score calculated as 6. b) CT image obtained with patient in prone position at RFA shows needle electrodes in tumor marked with green dots. c) CT image obtained with IV contrast material shows mass is no longer enhanced after 3 years (green arrow).

**Figure 2:**
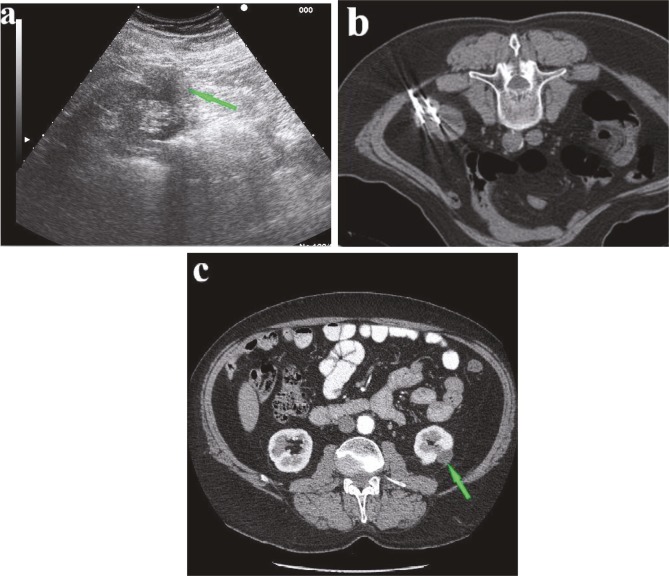
a) Seventy-year-old man with 4-cm biopsy-proven clear cell renal cell carcinoma (green arrow). RENAL nephrometry score calculated as 5. b) CT image obtained with patient in prone position shows cryoprobes in tumor. c) CT follow-up at 3 years demonstrates no evidence of tumor enhancement (green arrow).

During RFA, a 15-cm-long, 14-gauge RITA Starburst Talon thermal ablation electrode was used, in addition to a RITA model 1500X radiofrequency generator (RITA Medical Systems/Angiodynamics Inc., Latham, NY, USA) (Figures 1a–1c). Before starting the RFA process in 8 (17.7%) patients, in whom 8 (10.1%) lesions were located adjacent to solid organs, a 5% dextrose solution was injected into the adjacent kidney using an 18-G Chiba hydro-dissection needle to prevent organ damage. The Precise Cryoablation System and 4–6 Iceseed 17-gauge cryoablation needles (Galil Medical Company, Yoknaem, Israel) were used in the cryoablation process. A 10-min freeze-thaw cycle was applied twice after an appropriate number of probes (selected according to the dimensions of the lesion) were inserted at intervals of 1–2 cm (Figures 2a–2c).

One month after ablation, patients underwent dynamic contrast-enhanced CT (LightSpeed CT; GE Medical Systems, USA) or dynamic kidney MRI (Magnetom Verio; Siemens, Erlangen, Germany) and diffusion MRI studies. MRI was preferred as a follow-up imaging modality according to conditions such as patient preference, solitary kidney, and presence of impaired renal function tests. After months 1, 3, 6, and 12, control evaluations were obtained and then patients were followed at 1-year intervals.

Contrast retention in the initial follow-up imaging demonstrated a “residual tumor” in the ablated area. During the course of follow-up imaging, the appearance of new tumor foci at the ablative margin after local eradication was defined as “recurrence” [15].

All data were analyzed using SPSS 15.0 for Windows (SPSS Inc., Chicago, IL, USA).

## 3. Results

A total of 79 renal tumors in 45 patients were ablated. Mean lesion diameter was 2.2 (0.9–4.5) cm. Median follow-up was 18.5 (3–55) months (min–max). Tumor and ablation characteristics are presented in Table 1.

**Table 1:** Renal tumor and ablation characteristics.

	All ablations	Cryoablation	RFA
No. of renal tumors	79	15	64
Mean ± SD max diameter (cm)	2.2 ± 0.8	2.7 ± 0.8	1.7 ± 0.7
Percutaneous session	35	11	24
Intraoperative session	41	4	37
Laparoscopic session	3	-	3
Recurrence-free survival time (months)	34.8 (3–55)	44.6 (6–55)	28.6 (3–50)
Mean ± SD RENAL score	6.41 ± 1.7	6.40 ± 1.7	6.42 ± 1.7
RENAL score tumor complexity (%)			
Low (4–6)	46 (58.2)	8 (53.3)	38 (59.4)
Moderate (7–9)	29 (36.7)	7 (46.7)	22 (34.4)
High (10–12)	4 (5.1)	-	4 (6.2)

The distribution of lesions according to RENAL nephrometry score for complete ablation and residue/recurrence are presented in Table 2. The anterior/posterior parameter was not found to be significant in terms of outcomes. The initial average RENAL nephrometry score was 6.3 for completely treated tumors and 7.7 for tumors with residue/recurrence. The relationship between each group was significant (P < 0.016).

**Table 2:** Complete ablation and residue/recurrence relationship with RENAL nephrometry score.

		Complete ablation	Residue/recurrence
No. of renal tumors		72	7
Mean ± SD max diameter (cm)		1.8 ± 0.8	2.2 ± 0.8
Mean ± SD RENAL score		6.3 ± 1.6	7.7 ± 2.2
(R)adius		1.0 ± 0.1	1.0 ± 0.1
(E)xophytic/endophytic		2.0 ± 0.7	2.2 ± 0.9
(N)earness		1.6 ± 0.7	2.5± 0.7
(L)ocation		1.7 ± 0.8	1.8 ± 0.8
No. of anterior/posterior placements (%)			
Anterior		24 (33.4)	2 (28.6)
Posterior		32 (44.4)	5 (71.4)
No designation		16 (22.2)	-
RENAL score tumor complexity (%)			
	Low (4–6)	Moderate (7–9)	High (10–12)
Complete ablation	43 (54.4)	26 (33)	3 (3.8)
Residue/recurrence	2 (2.5)	3 (3.8)	2 (2.5)

The only major complication was pelvicalyceal system damage during RFA session in one patient, whose lesion was centrally located and whose RENAL nephrometry score was 9. The complication was repaired by a double J and drainage catheter in the Department of Interventional Radiology. Minor complications were treated conservatively. All complications, according to ablation procedure and RENAL nephrometry scores, are listed in Table 3.

**Table 3:** Complications.

	RFA	Cryotherapy
Major complications		
Pelvicaliceal system damage, n (%) – RENAL nephrometry score	1 (2.2) – 9	-
Minor complications		
Early impaired renal function tests, n (%) – RENAL nephrometry score	1 (2.2) – 8	-
Subcapsular hematoma, n (%) – RENAL nephrometry score	1 (2.2) – 6	-
Perirenal hematoma, n (%) – RENAL nephrometry score	2 (4.5) – 1st: 7 2nd: 6	1 (2.2) – 8
Skin burn, n (%) – RENAL nephrometry score	-	1 (2.2) – 7
Total, n (%)	5 (11.1)	2 (4.5)

As a superiority of ablative therapies to surgical methods, no significant difference was found between pre- and postablation in terms of the creatine levels for either ablation technique (P > 0.05).

The average recurrence-free survival (RFS) was 34.8 months, and RFS decreased with the size and number of lesions. In addition, while RFS was higher for exophytic lesions, it was quite low for parenchymal lesions and especially those with central location. The differences between exophytic, parenchymal, and centrally localized lesion groups were statistically significant (P < 0.001). The effects of risk factors on RFS are listed in Table 4.

**Table 4:** The effect of risk factors on recurrence-free survival by single-variable Kaplan-Meier survival analysis.

	RFS time,mean (95% CI*)	P-value
Lesion number		0.123
Single	48.1 (42.6–53.7)	
≥2	17.4 (10.0–24.7)	
Lesion size		0.140
<3 cm	47.7 (40.9–54.6)	
≥3 cm	37.4 (21.2–53.6)	
Lesion localization		0.001
Exophytic	50.8 (45.3–56.3)	
Parenchymal	37.1 (29.6–44.6)	
Central	10.5 (0.0–21.9)	
Ablation method		0.840
Cryotherapy	44.6 (34.0–55.1)	
RFA	28.6 (23.6–33.5)	

*CI: Confidence interval.

Patients treated with cryotherapy had higher survival rates than those treated with RFA. In the statistical study comparing multiple variables, the least effective factor on the RFS rate was ablation method; therefore, no subgroup analysis was performed statistically at this point for RENAL nephrometry score. The most notable risk factors for residue/recurrence were lesion size and location.

## 4. Discussion

Clinically, quite successful oncological results are obtained by nephron-protective surgeries for the treatment of tumors during the T1a stage [16,17]. The use of ablative methods is gradually increasing because these methods are less invasive and are associated with low complication rates. Hence, there is a need to develop a standardized method of anticipating complications and deciding on appropriate therapy. In this study, we used the RENAL nephrometry scoring system to prospectively evaluate RFA and cryotherapy in selected cases of renal cell carcinoma in terms of oncologic outcomes.

The RENAL nephrometry score is based on anatomical markers of the kidneys, as well as the locations and sizes of tumors [10,18,19]. The study with the largest patient series for the RENAL nephrometry scoring system was published by Schmit et al. [20]. An increase in the score is linked to an increase in recurrence and complication rate [11,21,22]. In our study, scores were higher in patients with residue/recurrence than those who underwent complete ablation. Furthermore, they were higher in patients who developed complications. However, no significant relationship was found between the nephrometry scores of lesions treated via RFA and cryotherapy. Accordingly, we assert that nephrometry scores can be considered a guide for surgeons and interventional radiologists in predicting the success of the preferred therapy but not for the type of ablation. At this point, studies to compare moderate-score and especially high-score lesions in terms of ablative and surgical treatment approaches may be useful in the future.

A previous study reported RFS rates of 87% and 90.6% and complication rates of 6% and 4.9% for RFA and cryotherapy, respectively [23]. It is generally accepted that the residue/recurrence and complication rates of RFA are higher than those of cryotherapy. Our results support these findings, as RFS was lower for lesions ablated with RFA than those treated with cryotherapy. The efficacy of RFA may decrease due to “heat-loss effect” in lesions located closer to the hilus and adjacent to vascular structures wider than 1 cm. Owing to the cryoresistance of the collecting duct system in these patients, cryotherapy should be the preferred ablation method [14,24]. 

A number of factors affect the survival rate in patients who receive ablative treatment and the most important prognostic factor is considered to be the size of the lesion [25,26]. In addition, tumor location is an important factor that may influence the success of ablation therapy [27,28].

We also observed that complications developed mostly in centrally and substantially parenchymal localized lesions. It was noted that these lesions were in the moderate or high risk group according to the RENAL nephrometry score.

Our study had some limitations. Fewer lesions were treated with cryotherapy than with RFA. Moreover, the presence of patients who had an excessive number of tumors related to hereditary renal disease and therefore had residue/recurrence in the RFA group led to heterogeneity and made statistical analyses of this group more complicated. In addition, patients may have been followed after treatment at other institutions, limiting the accuracy of data regarding recurrences after discharge.

In conclusion, for the management of renal masses, nonsurgical minimally invasive modalities such as RFA and cryotherapy are effective and safe alternatives to surgery in experienced hands. The RENAL nephrometry scoring system allows standardized evaluation of renal masses and the success of ablative therapies. Possible complications and oncological results can also be anticipated with this system. The number of studies comparing the efficacy of renal nephrometry scoring in RFA and cryoablation procedures in the literature is limited. It is emphasized that RENAL nephrometry scoring predicts the success of the preferred treatment procedure, but not the type of ablation.
